# Extracellular vesicle production in Gram‐positive bacteria

**DOI:** 10.1111/1751-7915.13956

**Published:** 2021-10-24

**Authors:** Scott N. Dean, Meghna Thakur, Joseph R. Spangler

**Affiliations:** ^1^ US Naval Research Laboratory Center for Bio/Molecular Science and Engineering Washington DC USA; ^2^ College of Science George Mason University Fairfax VA USA

## Abstract

This is a highlight on the article ‘Extracellular vesicle formation in *Lactococcus lactis* is stimulated by prophage‐encoded holin‐lysin system’ by Yue Liu, Eddy Smid and Tjakko Abee.

## Graphical abstract

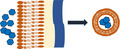

Extracellular vesicles (EVs) are secreted proteinaceous lipid bilayers derived from the membranes of bacteria and eukaryotic cells. Although the production of outer membrane vesicles (OMVs) from Gram‐negative bacteria has been thoroughly investigated over the past few decades, the origin, mechanism of formation and release, and other characteristics of EVs produced by Gram‐positive bacteria is less well understood (Brown *et al*., 2015). The formation of Gram‐negative OMVs has been extensively investigated in mechanistic studies and is generally described as forming via blebbing from the outer membrane caused by a number of contributing factors including reduction in crosslinking to peptidoglycan (Jan, 2017). Conversely, EVs secreted by Gram‐positives encounter additional obstacles from their origin at the cytoplasmic membrane to their arrival in the extracellular environment, namely the proteoglycan cell wall and other surface features (Fig. 1).

**Fig. 1:**
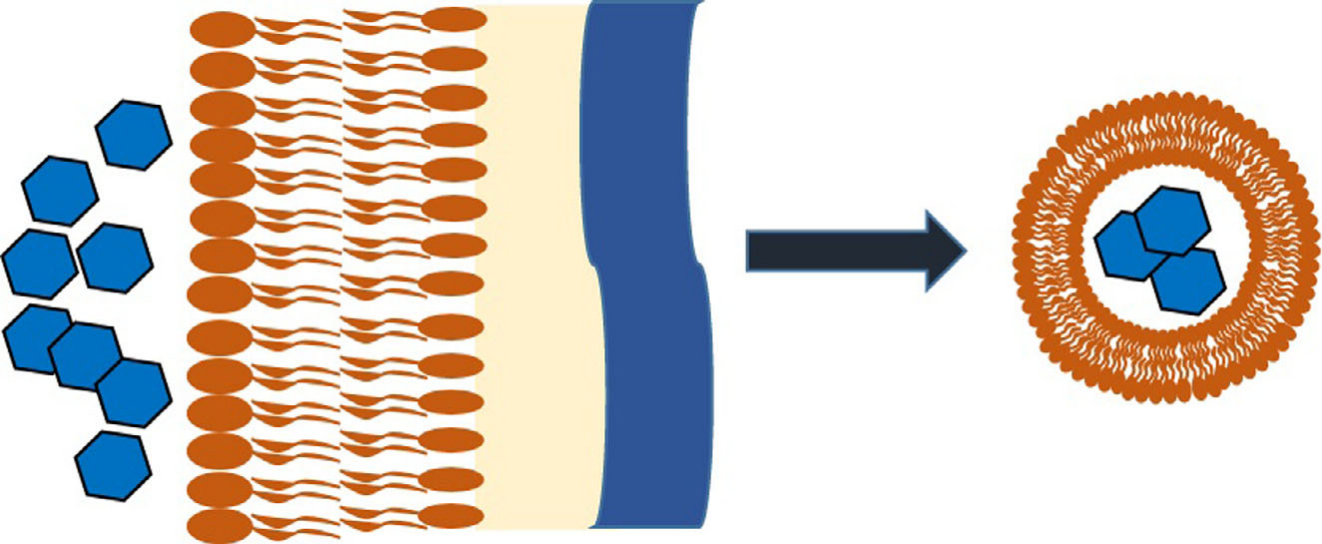
Schematic of extracellular vesicles produced by Gram‐positive bacteria.

The intricacies of vesiculogenesis in Gram‐positive have begun to be interrogated. Genetic control of EV production has been investigated in a number of species, where mutation of certain genes including broad gene regulators such as *sigB* and two‐component systems have been shown to significantly impact both EV production and characteristics (Briaud and Carroll, 2020). In *Staphylococcus aureus*, vesicle production was shown to depend on the production of amphipathic, alpha‐helical peptides called phenol‐soluble modulins that disrupt the membrane and promote EV formation (Wang *et al*., 2018). Release of EVs from the cell has been demonstrated in some species to depend on lipid composition, where dissimilarities between phospholipids in EVs and the parent bacteria suggest that production and release may occur at specific locations on the membrane. In many Gram‐positives, mass spectrometry proteomic analyses of EVs have shown the presence of penicillin‐binding proteins and autolysins suggesting their cell‐wall modification role is connected to vesicle release (Briaud and Carroll, 2020). Therefore, the current scheme for Gram‐positive EV production involves a complicated interplay of these mechanisms in addition to cell‐wall degrading enzymes allowing their release (Liu *et al*., 2018).

Despite the apparent hurdles to their production, a wide array of Gram‐positives have been demonstrated to produce EVs, including *S. aureus, Listeria monocytogenes, Streptococcus pneumoniae, Clostridium perfringens* and *Bacillus anthracis* as well as probiotic species *Lactobacillus acidophilus* and *Lactobacillus plantarum* among others (Dean *et al*., 2019; Bose *et al*., 2020). These EVs have begun to be well characterized over the past two decades, including early studies that reported proteomics profiling of the membrane‐bound and lumen‐carried proteins in various bacteria that have highlighted some potential mechanisms for Gram‐positive EV production, including the presence of peptidoglycan hydrolyase in EVs from *S*. *aureus* and phage‐associated endolysin from *S. pneumoniae* (Lee *et al*., 2009; Resch *et al*., 2016). In another interesting study, Toyofuku *et al*. (2017) found that *Bacillus subtilis* EV release was enabled by a prophage‐encoded endolysin.

The EV contents have begun to be viewed in the context of interaction with other cells and other functions in the Gram‐positive’s environment. In addition to proteins, Gram‐positive EVs have been shown to carry diverse cargo, including DNA that can be horizontally transferred between different species (Cao and Lin, 2021). In studies on their role in virulence, *B. anthracis* vesicles have shown to deliver their cargo – which include toxin components that are involved in cytotoxicity – to macrophages by phagocytosis or direct fusion (Wolf and Rivera, 2012). In opposition to the virulent effects of EVs originating from pathogens, the vesicles produced by probiotic bacteria have been implicated in protection against infection (Caruana and Walper, 2020), where, in one example, EVs of *Lactobacillus* species were shown to inhibit HIV‐1 infection of several human tissues *ex vivo* (Nahui Palomino *et al*., 2019). In another example, *Lactobacillus paracasei* vesicles significantly decreased the intestinal inflammatory response to lipopolysaccharide (Choi *et al*., 2020). This study is particularly interesting when considering the yet uncharacterized Gram‐positive EV architecture, as these EVs potentially maintain extracellular polysaccharides that have been shown to have health‐beneficial activities (Spangler *et al*., 2021). These anti‐inflammatory, anti‐infection and other positive effects of probiotic EVs are therefore of particular interest in the context of biotechnology and the emerging field of engineering probiotic bacteria (Spangler *et al*., 2021), where probiotics and the vesicles they produce can be engineered to further include therapeutic biomolecules or display characteristics favourable to vaccinations.

In the highlighted paper by Liu *et al*., they report investigations into the questions of vesiculogenesis in *Lactococcus lactis* EVs. In looking at the EVs produced by the lysogenic *L. lactis* strain FM‐YL11 they saw that prophage‐inducing conditions increased the production of EVs by greater than 10‐fold. They confirmed the role of the holin‐lysin system by creation of a prophage‐encoded holin‐lysin knock‐out mutant and prophage‐cured mutant which showed stable low‐level of vesicle production. Following further experimentation, including proteomic analysis of the EVs and amazing transmission electron microscopic images showing the presence of phage heads within the *L. lactis* vesicles, the group clearly demonstrated the function of the prophage‐encoded holin‐lysin system. Critically, the described system can be applied in the production of vesicles from other Gram‐positives, including other probiotic species (Liu and Smid, 2021). In the context of probiotic engineering, the findings by Liu *et al*. are particularly exciting as they enable both hypervesiculation and controlled production of the EVs, supplying a valuable engineering tool for a field where tools are currently lacking.

Future work stemming from the work of Liu *et al*. and other groups will likely further elucidate the questions surrounding Gram‐positive vesiculogenesis, the contents of EVs and their functions. The mechanism reported by Liu *et al*. could be widely applied among Gram‐positives, and probiotic bacteria in particular, and therefore deserves further investigation. These results may serve as a starting point for synthetic biologists to work on a means of control for EV production from probiotic species such as *L. lactis* and species of *Lactobacillus*. An engineered controllable, inducible system could allow for increased production of EVs for therapeutic or other biotechnology purposes, depending on their contents. While mysteries concerning the underlying mechanisms of Gram‐positive EVs remain unexplained, especially in the area of EV biogenesis, explanations of this component of the process, such as this report by Liu *et al*., will likely further encourage interesting new work that has clear applications in medicine and biotechnology.

## Conflict of interest

None declared.
